# Therapeutic Applications of Azo Dye Reduction: Insights From Methyl Orange Degradation for Biomedical Innovations

**DOI:** 10.7759/cureus.69952

**Published:** 2024-09-22

**Authors:** Sana Qausain, Mohd Basheeruddin

**Affiliations:** 1 Biochemistry, Jawaharlal Nehru Medical College, Wardha, IND

**Keywords:** azo dye, biocatalysts, biomedical applications, biosensors, drug delivery, enzymatic degradation, methyl orange, nanomaterials, therapeutic agents

## Abstract

This paper emphasizes the possible application of methyl orange reduction as a therapeutic technique, highlighting the potential of azo dye reduction in biomedical fields. The generally used azo dyes are toxic and carcinogenic; hence, they implicitly threaten the environment and health. The degradation of methyl orange, a famous example of azo dyes, is used to describe the degradation process for other azo dyes. This work discusses the ability of different methyl orange degradation methods, focusing on biocatalysts and nanomaterials, among the methods that identified enzymatic degradation with azoreductase enzymes as the method that quickly breaks down azo dyes under mild conditions as the most appropriate method, as well as its specificity as environmentally friendly. Moreover, metal nanoparticles such as silver and gold impellers increase the reducing efficiency because they offer a pivotal surface for the reduction reactions that undergo electron transfer. The complete breakdown of methyl orange is essential in biomedical usage. The strategies for treating azo dye reduction can be extended to next-generation drug delivery systems (DDS), biosensors, and therapeutic agents. Organisms involved in degradation can be functionalized to selectively degrade specific cells or tissues, thus presenting a new targeted therapy. Knowledge of degradation pathways and non-toxic products is essential in creating programs that build better and more efficient therapeutic agents. This work endeavors to illustrate the development of enzymatic and nanomaterials-based approaches to achieve sustainable azo dye decolorisation to open the gateway to developing other biomedical applications that tend to promote environmental and health-friendly solutions.

## Introduction and background

Azo dyes, which are brightly coloured and used mainly in the textile, pharmaceutical, and food industries, are dangerous due to their toxicity, carcinogenicity, and non-biodegradability, resulting in pollution. Methyl orange, a colourless substance in solution, is a widely used pH indicator, dyeing agent, and azo dye [1]. Several techniques are widely used in removing methyl orange dye that poses threats to the environment, and these include chemical reduction, photo-degradation, and enzymatic degradation (see Table 1).

**Table 1:** Overview of azo dyes and their degradation Ref. [1]

Aspect	Details
Class of dyes	Azo dyes
Characteristics	Vivid colors, use in textile, pharmaceutical, and food industries
Environmental and health hazards	Toxic and carcinogenic properties
Common laboratory use	Methyl orange- pH indicator and dyeing agent
Significance of degradation	Environmental concerns
Degradation methods	Reduction, photodegradation, enzymatic degradation
Enzymatic degradation	Catalyzed by azoreductases
Azoreductases	Catalyze the azo bond (-N=N-) in dyes
Nanomaterials in degradation	Use of silver and gold
Broader implications	Therapeutic agents, drug delivery systems, and biosensors

Azoreductases are enzymatic degradation specific and ecologically friendly, being mild about temperature. Further, nanomaterials such as silver and gold nanoparticles improve the degradation process by offering space for electron transfer. The knowledge of methyl orange degradation mechanisms will help design superior therapeutic agents, drug delivery systems (DDS), and biosensors, which are some of the broader biomedical importance of azo dye reduction [2].

## Review

Classification of azo dyes

Azo-based dyes are subdivided by the number of azo linkages in the molecule; there are monoazo, disazo, trisazo, polyazo, and azoic dyes. The color index (CI) classification of azo dyes is in series numbering 11000-39999 based on chemical structure, which is depicted in Table 2. This color index number, as formulated by the Society of Dyers and Colorists, is used for dye categorization [3].

**Table 2:** Classification of azo dyes according to the color index (CI) Source: Ref. [3]

Chemical class	CI no
Monoazo	11000–19999
Disazo	20000–29999
Trisazo	30000–34999
Polyazo	35000–36999
Azoic	37000–39999

Mechanisms of azo dye reduction

Azo dye levelling minimizes toxicity and increases the biodegradability of a synthetic dye by breaking the azo bond (-N=N-) in the dye molecule. We now discuss these in more detail, including some of the main pathways that enable this reduction, including enzymatic processes, chemical reduction, and microbial degradation [4].

Chemical Reduction

This study considered the chemical reduction in which azo dyes were treated by reducers such as sodium dithionite, and as a result, the azo bond (-N=N-) was disrupted, and the dyes were turned into less toxic and highly biodegradable substances. This method is easy to perform and can be used to cleave azo dyes; however, it is prone to perform under harsh conditions such as high temperatures or in acidic solutions [5]. Third, chemical reduction can produce second contaminants, which are also dangerous to the environment and man's health. Because of the need for precise reaction conditions and the need for control of the byproducts, the generality of this approach is limited; nonetheless, it helps remove azo dyes [6].

Photochemical Reduction

Azon hydrolysis uses light energy to stimulate bound dye molecules from the ground state to an excited state, in which electron loss will result in the cleavage of the azo bond (-N=N-). The method can be significantly effective, provided it is operated under the excitation and reduction processes corresponding to the light source's specified wavelengths. However, efficient the photochemical reduction process is, its usefulness is confined because it requires controlled light sources and conditions [7]. The need for specialized equipment and the critical aspect of light penetration means that large-scale or variable environments where the technique may prove helpful will be limited in application. Nevertheless, photochemical reduction is still viable for the specific and effective removal of azo dyes [5].

Enzymatic Reduction

Enzymatic reduction of azo dyes utilizes azoreductase enzymes, which catalyze the cleavage of the azo bond (-N=N-) by transferring electrons from cofactors such as nicotinamide adenine dinucleotide (NADH) or nicotinamide adenine dinucleotide phosphate (NADPH) to the dye molecule.

This process occurs under moderate and non-hazardous conditions, often at near-neutral pH and a moderate temperature [8]. Azoreductases have been isolated from different microbial sources, bacteria, and fungi, which make them general catalysts for azo dye reduction in different ecosystems and industries. Due to the maturity in the degradation of azo bonds, they exhibit high selectivity that leads to efficient dye degradation while minimizing the production of byproducts thus ensuring environmental friendly [9].

Nanoparticle-Assisted Reduction

Some approaches to reducing azo dyes include using metal nanoparticles, mainly silver and gold, that work under photocatalytic reduction to provide the needed surface to catalyze electron transfer reactions. These nanoparticles have active areas where electrons can quickly transfer to the azo bonds (-N=N-), breaking them into less hazardous compounds. The azo dye reduction has been known to be enabled through the use of metal nanoparticles, where the efficiency of the process can be engineered through different methodologies. For instance, the identified nanoparticles are arguably more effective, silver and gold nanoparticles, because of their high surface-to-volume ratio and preferential electronic properties [10]. The catalysts can enhance the reduction process under mild reaction conditions and, as such, can be applied in laboratory research and large-scale work such as water treatment and environmental management. In addition, nanoparticle-assisted reduction advances the research on new and efficient methods to combat azo dyes’ detrimental environmental effects as part of a more environmentally friendly and safer process [11].

Still, these mechanisms are helpful and can be adapted to various uses, for example, water treatment or sophisticated biotechnology applications. Particularly worthy of consideration is using enzymatic and nanoparticle-assisted methods to reduce azo dyes at once [4].

Applications of azo dye reduction in biomedical science

Azo dye reduction strategies offer potential in DDS because one can design intelligent materials that will deliver drugs in a controlled manner. From the cleavage of azo bonds (-N=N-) under certain physiological conditions such as pH or enzymatic activity in Table 3, scientists and engineers design drug carriers that are stable during circulation but release their payloads on reaching the tissues or cells of interest [5].

**Table 3:** Various uses of azo dye reduction in biomedical engineering Source: Ref. [5]

Application	Description	Key Focus
Drug Delivery Systems	Utilizes azo bonds as triggers for controlled drug	Targeted delivery, enhanced efficacy, reduced side effects
Tissue Engineering	Incorporates azo dyes into scaffolds	tissue regeneration, spatial-temporal control
Environmental Health	Applies azo dye reduction to detoxify and mitigate environmental pollution	Bioremediation, sustainable degradation, regulatory compliance

DDS

In biomedical applications, azo dye reduction methodologies present innovative solutions for advancing DDS with enhanced functionality and efficacy. By harnessing azo bonds (-N=N-) as triggers for drug release, researchers can engineer intelligent DDS that respond to specific physiological conditions, such as pH or enzymatic activity, prevalent in disease micro-environments [12].

Mechanisms and design: Most applications of azo bonds mentioned above are considered in the context of their utilization as responsive elements in DDS, where azo dyes are incorporated into the DDS matrix. They are supposed to be stable during circulation in the human body. They can be cleaved selectively upon certain stimuli such as pH value (acidic pH) or enzymes, thus releasing the drug at the disease site. This is made more accessible by developing responsive nanoparticles and polymers conjugated with azo dyes. These sophisticated systems can control drug release rates and responses to the stimulant in the surroundings, such as the tumor’s CYA environments or the agencies overexpressed in inflamed tissues, to enhance treatment efficiency and selectivity [13].

Advantages in biomedicine: Azo dye-mediated DDS has emerged as one of the most promising approaches, and the enrichment of DDS for targeted delivery of drugs to the specific tissues or cells of the body, thus moderating the impact of the drug on the rest of the body system. These systems help improve the therapeutic drug release profiles and patient compliance through controlled and sustained release. Moreover, since azo dyes are flexible for modification to achieve various functional groups and DDS can be developed to respond to the different stimuli related to the diseases, the multifunctional systems should offer diverse therapeutic strategies, leading to better therapeutic outcomes and fewer side effects [14].

Tissue Engineering

In the context of biomedical applications, azo dye reduction techniques offer innovative solutions for advancing tissue engineering approaches with enhanced functionality and efficacy. By utilizing azo bonds (-N=N-) as triggers for structural modifications, researchers can engineer innovative scaffolds and biomaterials that respond to specific physiological conditions, such as pH or enzymatic activity, prevalent in diseased tissues or during regeneration processes [15].

Mechanisms and design: Substances known as azo dyes, incorporated within scaffold materials and biomaterials, are responsive agents that only get cleaved under specific conditions and, hence, can create structural changes and release biomolecules at particular locations within the TE constructs. This functionality is further enhanced by developing different responsive nanoparticles and polymers functionalized with azo dyes to control the kinetics of scaffold degradation and biomolecule release. They can be designed to respond to the specific conditions they experience in simple tissue environments, such as pH when operating under inflammatory conditions or the activity of tissue remodelling enzymes when in tissue regeneration phases to enhance tissue engineering performances [3].

Advantages in biomedicine: Specifically, the azo dye-based tissue engineering strategies significantly improve tissue repair as the growth factors, cytokines (e.g., interleukin-6 (IL-6), tumor necrosis factor-alpha (TNF-α), and transforming growth factor-beta (TGF-β)), and other bioactive agents (e.g., vascular endothelial growth factor (VEGF), fibroblast growth factor (FGF), and bone morphogenetic proteins (BMPs)) can be released in a controlled manner. These systems provide spatial and temporal regulation of therapeutic agent delivery through proper profiling of scaffold degradation rates, thus guaranteeing the availability of bioactive molecules in appropriate locations and time for efficient tissue repair. The functionality of azo dyes also creates multi-stimuli responsive scaffolds that may be sensitive to various tissue-specific signals, which is useful for tissue engineering applications that are versatile enough to address various regenerative requirements [16].

Environmental Health

**Figure 1:**
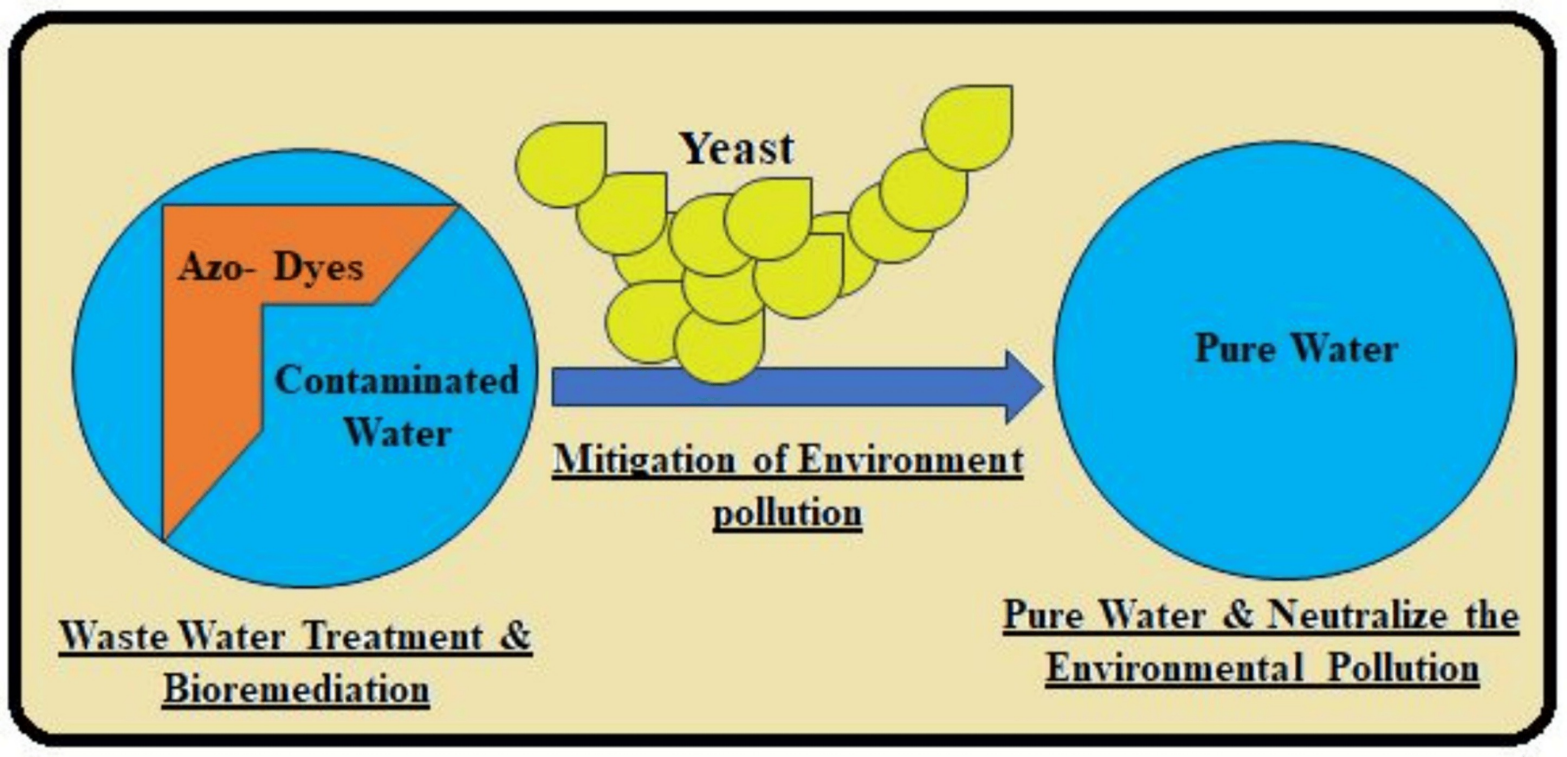
Utilizing azo bonds for the degradation process to detoxify and mitigate yeasts Image credits: Sana Qausain

In biomedical applications, azo dye reduction methodologies offer innovative solutions for addressing environmental health concerns associated with the widespread use and disposal of azo dyes. By utilizing azo bonds (-N=N-) as triggers for degradation processes, researchers can develop environmentally friendly approaches to detoxify and mitigate yeast. Figure 1 shows the impact of these hazardous compounds on ecosystems [5].

**Figure 2:**
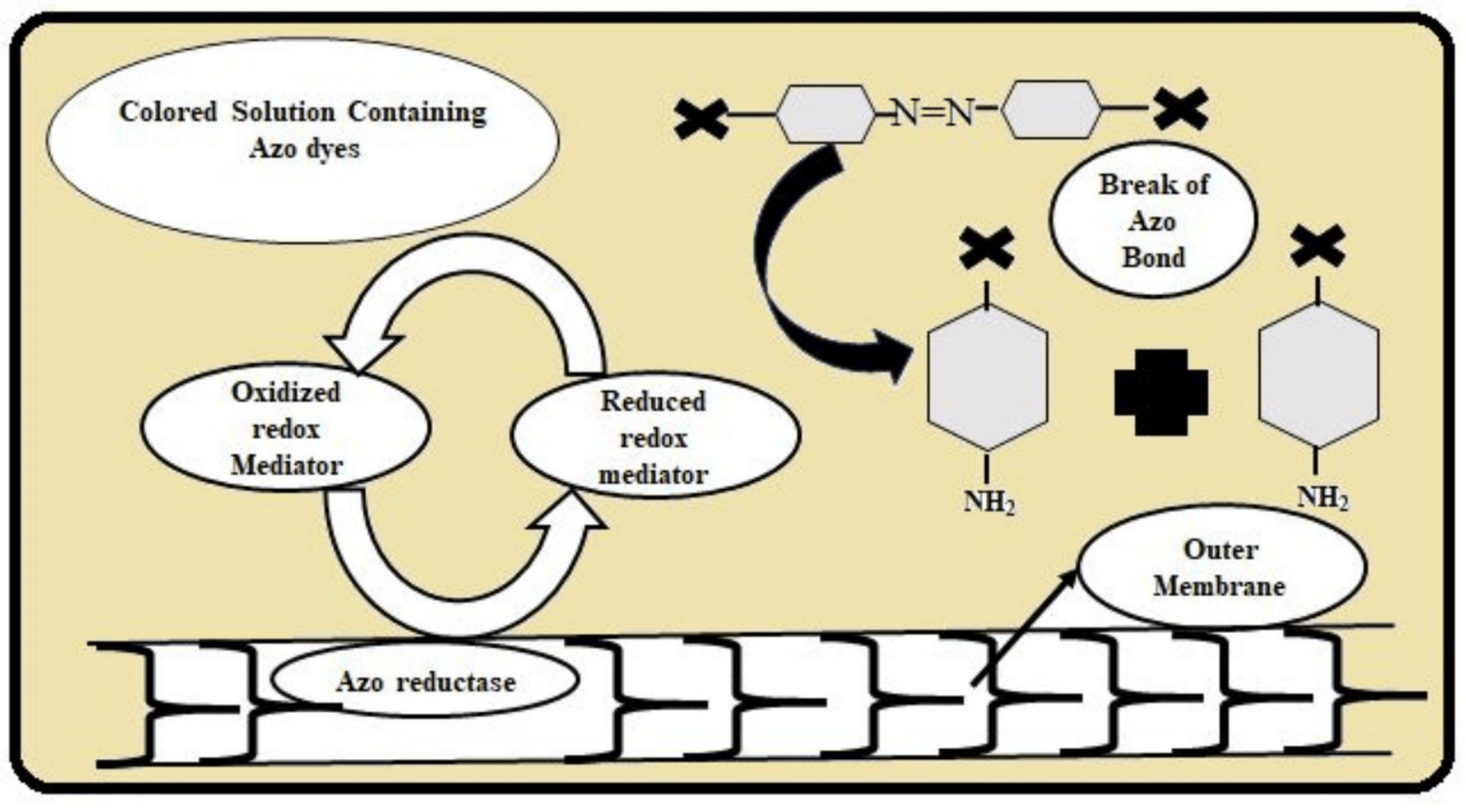
Mechanisms of azo dyes Image credits: Mohd Basheeruddin

Mechanisms and design: Azo dyes, when subjected to enzymatic or chemical treatment, for example, the cleavage of the azo bond (Figure 2), reduce into harmless products and therefore make it easier to eliminate these dyes from the polluted water and environment. This approach is especially beneficial in bioremediation techniques where enzymes such as azoreductases, the enzymes isolated from microbes, facilitate the cleavage of the azo bonds under mild conditions. The type of degradation is enzymatic, which does not require substantial energy input. At the same time, the products formed are non-hazardous to the environment, providing a solution to a real-world problem of azo dye pollution [9].

Advantages in biomedicine: Azo dye reduction technologies are essential to environmental conservation since they decrease the bioavailability of azo dyes to the environment, reducing their persistence and toxicity. These technologies help in the degradation of the azo dyes into their simplest forms, enhancing water and soil purification. Hence, this paper demonstrates how safe disposal and azo dye reduction minimizing treatments' effect on the environment (Figure 1) helps meet the regulatory standards in wastewater treatment [1].

Recent research advances

**Figure 3:**
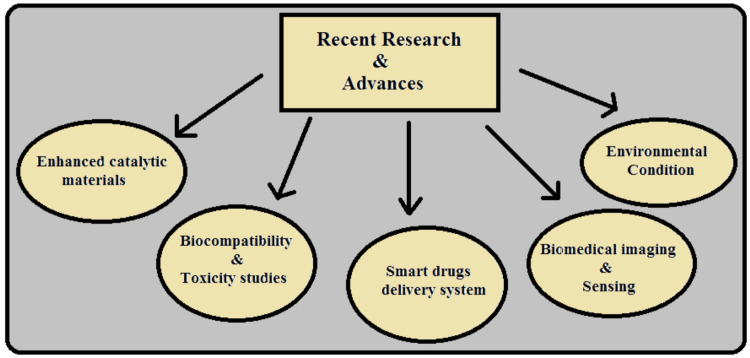
Recent research advances in azo dye reduction and its biomedical implications Image credits: Sana Qausain

Recent research has significantly advanced the therapeutic potential of azo dye reduction, especially in the degradation of methyl orange, with notable implications for biomedical applications (see Figure 3). They include synthesizing new catalytic materials, biomedical compatibility, antimicrobial and antibacterial properties, enzyme assays, cancer therapies, targeted drug delivery, and investigations of biomedical imaging and environmental scanning [17].

Enhanced Catalytic Materials

Scientists have synthesized new catalytic materials such as metal-organic frames and hybrid nanocomposites that show higher catalytic activity in degrading MO. However, there has yet to be a report of its application in the degradative study of methyl orange using Cu/Al layered double hydroxide (LDH). These materials introduce enhanced catalytic activity and stability, making them plausible materials for use in the biomedical field and the environment [18].

Biocompatibility and Toxicity Studies

Researchers have also explored the biocompatibility and toxicity profiles of the products of azo dyes. Consequently, efficient analytical tools coupled with cell culture systems have been used to adequately establish the safety profile of degraded products for their biomedical application [19].

Smart DDS

Azo dye reduction principles are also incorporated into intelligent DDS, where therapeutic agents are released on the breakdown of azo bonds. This strategy enables excellent regulation of the drug release rate, thus improving the drug’s delivery and effectiveness [20].

Biomedical Imaging and Sensing

Scientists have focused on the optical properties of the azo dyes for use in biomedical imaging and sensing. These studies have shown that it is possible to employ azo dyes for diagnostic purposes or as probes identifying particular biomolecules or pathophysiological status [21].

Environmental Conditions

Apart from biomedical fields, newer research has extended to environmental applications of the technology and removing the azo dye in wastewater treatment using eco-friendly techniques. Fundamental mechanisms of the advanced oxidation processes and enzymatic degradation pathways have been enhanced to manage environmental pollution [22].

These recent inventions show that more and more scientists are exploring the tremendous possibilities of azo dye reduction technologies for enhancing the biomedical and environmental sciences. New research ideas are directed to improve these technologies for practical application in the future, with prospects of how to deal with issues like scalability, biocompatibility, and regulatory barriers [23].

Future perspectives

The prospects for further consideration of azo dye reduction as a therapeutic intervention for the breakdown of alternative oxidase (AOX) and specifically methyl orange with particular regard to biomedicine include multiple aspects.

Advanced Materials Development

Future studies will probably focus on creating more elaborated catalytic materials with higher activity and selectivity about azo dyes’ degradation. This also concerns the development of brand-new nanomaterials such as metal-organic frameworks (MOF) and hybrid nanoparticles in terms of enhanced catalytic performance and selectivity under different environmental and physiological situations [24].

Biomedical Applications Expansion

**Figure 4:**
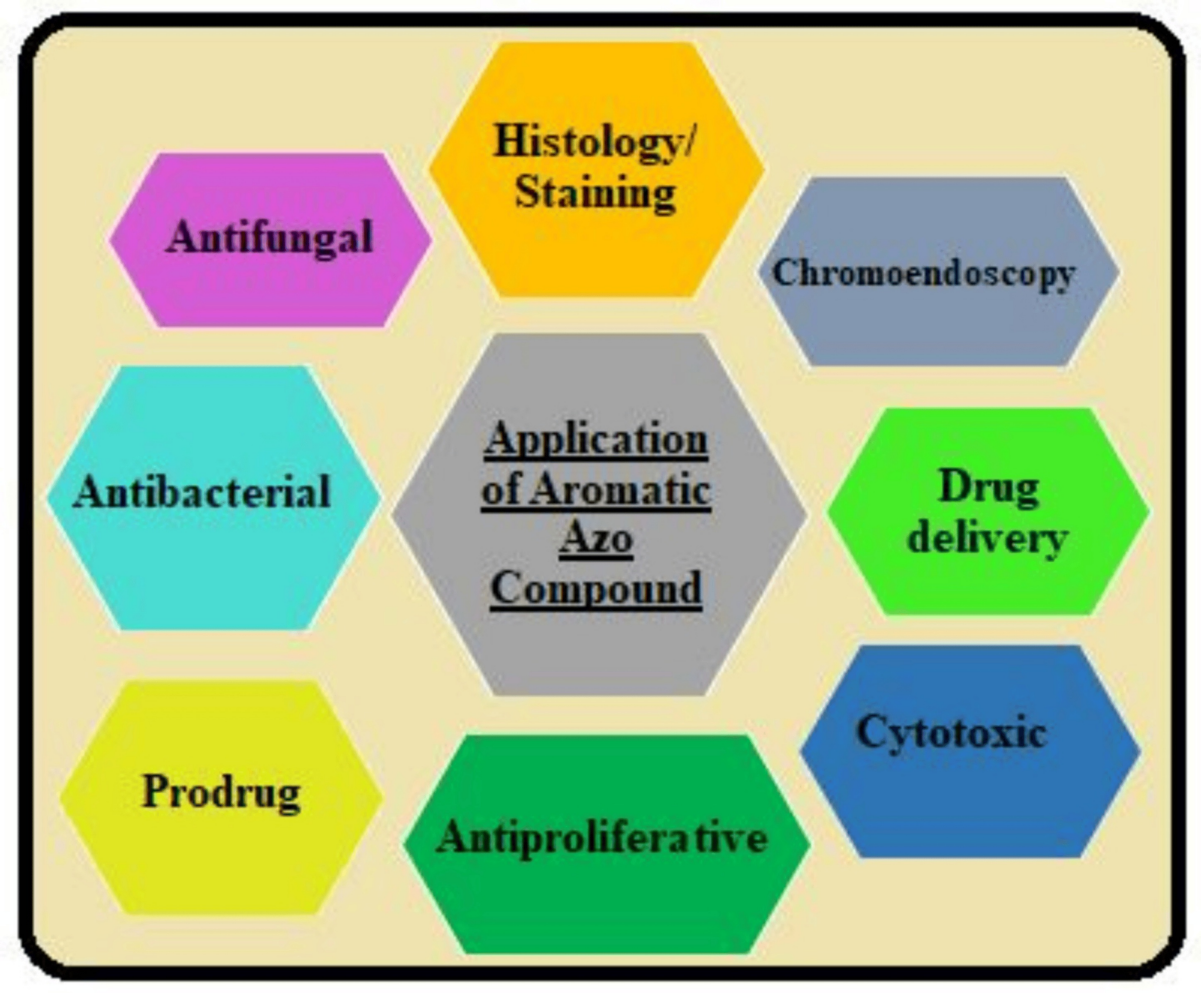
Application of aromatic azo compound Image credits: (Sana Qausian)

As shown in Figure 4, azo dye reduction of the aromatic azo compound used in this study can be applied to controlling drug release via hydrogels, etc. The focus of future research may extend to tissue engineering, where the azo dyes can be incorporated into scaffolds that can allow the regulation of degradation and controlled release of biomolecules for tissue healing and regeneration [25].

Biocompatibility and Safety Assessments

Further studies will concern the evaluation of the biocompatibility and toxicity of the products that result from the degradation of azo dyes for biomedical applications. Therefore, comprehensive studies, clinical and basic, employing sophisticated tools and rigorous testing on in vitro and vivo models, are inevitable in advancing these technologies to apply them safely in the clinic [5].

Smart and Responsive Systems

Specifically, creating intelligent DDS that respond to the azo bonds as the signal for drug release will remain a current interest. It is also possible to extend DDS concepts to programs that behave actively in response to certain stimuli and situations to attain definite adaptation of drug release kinetics to required disease environments or the individual’s condition [26].

Environmental and Regulatory Considerations

With the increasing improvement of the concepts of azo dye reduction technologies, the concern shall shift to sustainability and legal compliance. Further, research activities will probably be directed to fine-tuning these processes for long-term application in cleaning the environment and water pollution control to meet the growing international concern about environmental pollution and using scarce resources [22].

Integration with Imaging and Sensing Technologies

Biomedical imaging and sensing methods may be combined with azo dye-based technologies. In future studies, it will be valuable to look at the uses of these nanomaterials in imaging, as contrast agents or biomarkers, or in detecting and monitoring therapeutic outcomes, thereby adding to diagnostic and monitoring aspects in clinical practice [27].

To sum up, the future of azo dye reduction in biomedical applications, particularly in methyl orange degradation, has achieved an excellent potential for improvement in all these areas. The progress of interdisciplinary cooperation with scientific and technological expeditions will advance, extending into practical use and contributing to new methods and approaches to healthcare and the environment.

## Conclusions

The decolorization of azo dyes such as methyl orange has been proven to possess enormous bibliotherapeutic benefits in biomedical science. Since azo bonds (-N=N-) undergo stimulus-sensitive cleavage, a more sophisticated DDS can be developed that responds directly to changes in one or other disease conditions, such as pH or enzyme activity. This approach helps maximize the specificity and effectiveness of the drug treatments with the least amount of side effects on the body. The present work suggests more research is needed to improve the biocompatibility and safety of azo dye material for the intended medical use. In addition, the research on further application of azo dyes in tissue engineering and environmental deterioration provides valuable perspectives to solve health and environmental problems. Thus, these investigations underscore the twofold opportunity of azo dye technologies: enhancing patient-centered ethical care and developing environment-friendly technologies. These materials have the potential to improve the efficacy of therapeutic interventions and, at the same time, minimize environmental degradation, making them crucial in defining the future of health delivery systems.

In the future, collaborative studies are going to be crucial to turn these scientific discoveries into technologies and products that help to enhance human health and the state of the environment. Therefore, there exists a universal potential for developing new technologies for the reduction of azo dyes, remodeling the avenues of targeted DDS, and enhancing biomedical treatment options.
